# Treatment persistence of bictegravir/emtricitabine/tenofovir alafenamide and efavirenz + lamivudine + tenofovir disoproxil among HIV-1 patients newly starting treatment in Hunan Province in China

**DOI:** 10.1186/s12879-023-08359-w

**Published:** 2023-06-12

**Authors:** Cao Jing, Tang Wei, Wang Ning, Zheng Fang, Xiao Gang, Wang Xingzhi, Zhou Guoqiang, Wang Min

**Affiliations:** 1 Division of Infectious Diseases, the First Hospital of Changsha, Changsha, Hunan China; 2 Yidu Cloud Technology, Shanghai, China

**Keywords:** BIC/FTA/TAF, EFV + TDF + 3TC, Persistence, Treatment discontinuation, First-line therapy, China

## Abstract

**Background:**

Though bictegravir/emtricitabine/tenofovir (BIC/FTC/TAF) have been regulatory approved and included in the National Reimbursement Drug List in China, due to the affordability concern, generic version of efavirenz + lamivudine + tenofovir (EFV + 3TC + TDF) is still recommended as the first-line therapy in the clinical guideline and widely used in clinical practice. The aim of the study is to assess the persistence with first-line BIC/TAF/TAF and EFV + 3TC + TDF in newly treated HIV-1 patients in the real-world setting in Hunan Province in China.

**Methods:**

A retrospective analysis of the medical records of HIV patients initiating first-line antiretroviral therapy in the First Hospital of Changsha in January 1st, 2021-July 31st, 2022 was conducted. Persistence was assessed as the number of days on the therapy from the index until treatment discontinuation or end of data availability. Kaplan-Meier Curves and Cox Proportional Hazard models were used to evaluate the discontinuation rates. Subgroup analysis was performed excluding BIC/FTC/TAF patients with treatment discontinuation due to economic reason, and EFV + 3TC + TDF patients with a viral load > 500,000 copies/mL.

**Results:**

A total of 310 eligible patients were included in the study, with 244 and 66 patients in the BIC/FTC/TAF group and EFV + 3TC + TDF group, respectively. Compared with EFV + 3TC + TDF patients, BIC/FTC/TAF patients were older, more living in the capital city currently, and had significantly higher total cholesterol and low-density level (all p < 0.05). No significant difference was shown in the time to discontinuation between BIC/FTC/TAF patients and EFV + 3TC + TDF patients. After excluding BIC/FTC/TAF patients with treatment discontinuation due to economic reason, EFV + 3TC + TDF group were shown to have a significantly higher risk of discontinuation than BIC/FTC/TAF group (hazard ratio [HR] = 11.1, 95% confidence interval [CI] = 1.3–93.2). After further removing the EFV + 3TC + TDF patients with a viral load > 500,000 copies/mL, the analysis showed similar results (HR = 10.1, 95% CI = 1.2–84.1). 79.4% of the EFV + 3TC + TDF patients discontinued treatment due to clinical reasons, while 83.3% of the BIC/FTC/TAF patients discontinued treatment due to economic reasons.

**Conclusions:**

Compared with BIC/FTC/TAF, EFV + TDF + 3TC patients were significantly more likely to discontinue the first-line treatment in Hunan Province in China.

## Background

In China, HIV imposed a significant disease burden. The China Center for Disease Control and Prevention (CDC) reported 1.05 Million HIV/AIDS cases alive and 351 thousand cumulated death by end of 2020 [1]. From 2005 to 2019, the annually newly reported HIV/AIDS cases increased 15 times from 2,705 cases to 42,406 cases [2]. Specifically, the number of newly diagnosed HIV/AIDS cases among older people increased dramatically. The CDC reported that from 2007 to 2018, the incidence rate of HIV/AIDS among the population aged 60 years and above increased 10.3 times and 10.8 times among males and females, respectively [3]. The aging HIV/AIDS population indicates the needs for treatment regimens with better efficacy and safety profiles. .

Antiretroviral therapy (ART) has been introduced in the HIV treatment and significantly lowered the mortality and morbidity of HIV patients. The Chinese Guidelines for Diagnosis and Treatment of Human Immunodeficiency virus Infection/Acquired Immunodeficiency Syndrome (2021 edition) has recommended initiation ART in treatment-naïve patients with the regimen consisting of two nucleoside reverse transcriptase inhibitors (NRTIs) plus a third drug [4]. The third drug could be either a non-nucleoside reverse transcriptase inhibitor (NNRTI), or a boosted protease inhibitor (PI), or an integrase strand transfer inhibitor (INSTI). The single-table regimen (STR) is recommended. In the guideline, bictegravir/emtricitabine/tenofovir alafenamide (BIC/FTC/TAF), elvitegravir/cobicistat/emtricitabine/tenofovir alafenamide (EVG/c/FTC/TAF), dolutegravir/abacavir/lamivudine (DTG/ABC/3TC) and doravirine/tenofovir/lamivudine (DOR/TDF/3TC) were the four STR regimens recommended, among which, BIC/FTC/TAF and EVG/c/FTC/TAF have been included in the 2021 Edition National Reimbursement Drug List [5]. Nevertheless, mainly due to the affordability concern, multiple tablet regimens (MTRs) with generic drugs, especially the generic version of efavirenz + lamivudine + tenofovir (EFV + 3TC + TDF), are widely used in real-world clinical practice. In China, the national free ART program (NFATP) was launched in 2003 to help HIV patients to obtain the access to ART [6]. The first guideline for HIV/AIDS diagnosis and treatment was published in 2005 and since then, an increasing number of ARV drugs have become available to HIV patients free of charge [7, 8]. Generic version of EFV + 3TC + TDF has been recommended as the first-line therapy since the 2011 China National Guidelines for HIV/AIDS Diagnosis and Treatment, and is widely used in clinical practice in China [9].

Previous studies have shown that STRs had higher adherence and persistence compared with MTRs [10–13]. Nevertheless, limited studies have explored the persistence difference between STR and MTR in the real-world clinical practice in China. Since BIC/FTC/TAF and EFV + 3TC + TDF are the representatives of STR and MTR in China, respectively, the aim of the study is to compared the treatment persistence of BIC/FTC/TAF and EFV + 3TC + TDF in treatment-naïve HIV-1 patients.

## Methods

### Study design and data source

This is a retrospective database analysis using the electronic medical record data in the Department of Infection in First Hospital of Changsha to evaluate the persistence of ART regimens in treatment-naïve HIV-1 patients. The hospital is a tier 3 A hospital in Hunan Province, covering the majority of the treatment of HIV/AIDS patients in the province. The patients’ data collection started on January 1st, 2021 and ended on July 31st, 2022 with the last hospital visit. The study was conducted in accordance with the Chinese Ethical Guidelines for Medical and Health Research Involving Human Subjects. Ethical approval was required according to the guidelines.

### Study population

Adult patients diagnosed with HIV-1 infection between January 1st, 2021 and July 31st, 2022, with the prescription of BIC/FTC/3TC or generic version of EFV + 3TC + TDF as the first-line treatment, and have at least one hospital visit after prescription, were included in the study. The index date was the first time for patients prescribed with BIC/FTC/3TC or EFV + 3TC + TDF. Persistence was measured by the duration on the regimen, calculated from the index date until the last date when the regimen was prescribed. In clinical practice, BIC/FTC/3TC patients could switch to free ARTs because of the economic burden of BIC/FTC/TAF. Therefore, a subgroup analysis was performed excluding BIC/FTC/3TC patients with treatment switch due to affordability reason. Furthermore, since EFV is not recommended for patients with a viral load > 500,000 copies/mL in the 2021 Edition HIV/AIDS diagnosis and treatment guideline, a subgroup patient population was selected with a viral load ≤ 500,000 copies/mL.

### Statistical analysis

Continuous variables were reported with mean and standard deviation (SD). Categorical variables were reported with count (frequency) and percentage. T-test and chi-square test were used in between-cohort comparisons for continuous variables and categorical variables, respectively. The time to discontinuation of index regimen was estimated by Kaplan-Meier curves, with log-rank tests to assess the statistically significant difference between the two regimen groups. Cox proportional hazard models were used to assess the risk of discontinuation. Age group, gender, baseline viral load groups, baseline CD4 cell count groups, baseline total cholesterol, baseline low-density lipoprotein, education level, employment status and current residence were included as the covariates in the model. All statistical analysis were performed with SPSS® version 26.0 (IBM Corp., Armonk, NY). The statistical significance of all tests was assessed with a significance level of 0.05.

## Results

A total of 310 eligible patients were included in the study, with 244 and 66 patients in the BIC/FTC/TAF group and EFV + 3TC + TDF group, respectively. The demographic and clinical characteristics are described in Table 1. The mean ± standard deviation ages of patients with BIC/FTC/TAF and EFV + 3TC + TDF were 35.8 ± 13.6 and 31.4 ± 12.5 years, respectively (P = 0.015). There were more male patients than female patients (BIC/FTC/3TC group: 97.0%; EFV + 3TC + TDF group 94.2%) and nearly half of the patients (BIC/FTC/3TC: 45.3%; EFV + 3TC + TDF: 48.0%) have a viral load ≤ 50,000 copies/mL. There were significantly more patients in BIC/FTC/3TC (95.5%) group living in Changsha, the capital city of Hunan Province, than those in the EFV + 3TC + TDF (85.2%) group (p = 0.027). Patients in BIC/FTC/TAF group (35.9%) reported a higher proportion with a CD4 counts less than 200 cells per µL, compared with patients in EFV + 3TC + TDF (23.8%) group (p = 0.054). BIC/FTC/FAF group had significantly higher total cholesterol and low-density level, compared with EFV + 3TC + TDF group (both p < 0.05). There were no significant differences between BIC/FTC/TAF group and EFV + 3TC + TDF group in gender, education level, employment status, HIV-1 RNA concentration, CD4 counts, creatinine clearance, triglyceride, and high-density level (all p > 0.05).

**Table 1:** Demographic and clinical characteristics of EFV + 3TC + TDF and BIC/FTC/TAF treatment groups

Variable		EFV + FTC + 3TC		BIC/FTC/TAF		P-value
		N = 244		N = 66		
Age (years), mean (SD)		31.4 (12.5)		35.8 (13.6)		0.015
Gender, n (%)						
	Male	231	(94.7%)	64	(97.0%)	0.440
	Female	13	(5.3%)	2	(3.0%)	
Education, n (%)						
	Primary	12	(4.9%)	2	(4.5%)	0.661
	Secondary	86	(35.2%)	21	(31.8%)	
	Tertiary	146	(59.8%)	43	(65.2%)	
Employment status, n (%)						
	No formal work	76	(31.1%)	22	(33.3%)	0.413
	Formal work	117	(48.0%)	29	(53.0%)	
	Student & retirement	51	(20.9%)	15	(13.6%)	
Current residence, n (%)						
	Changsha	208	(85.2%)	63	(95.5%)	0.027
	Out of Changsha	36	(14.8%)	3	(4.5%)	
HIV-1 RNA (log10 copies/mL), mean (SD)		4.7	(0.7)	4.8	(0.6)	0.363
HIV-1 RNA concentration (copies/mL), n (%)						
	≤ 50,000	95	(48.0%)	29	(45.3%)	0.775
	50,000–100,000	51	(25.8%)	15	(23.4%)	
	100,000–500,000	38	(19.2%)	13	(20.3%)	
	> 500,000	14	(7.1%)	7	(10.9%)	
CD4 count (cells/µL), mean (SD)		326.2 (185.8)		317.8 (199.0)		0.753
	< 200	57	(23.8%)	23	(35.9%)	0.054
	200–349	87	(36.4%)	13	(20.3%)	
	350–499	59	(24.7%)	15	(23.4%)	
	≥ 500	36	(15.1%)	13	(20.3%)	
Creatinine clearance (µmol/L), mean (SD)		71.1	(11.2)	73.3	(16.2)	0.307
Triglyceride (mmol/L), mean (SD)		1.6	(1.1)	1.9	(1.5)	0.061
Total cholesterol (mmol/L), mean (SD)		4.2	(0.9)	4.5	(1.1)	0.010
LDL (mmol/L), mean (SD)		2.8	(0.7)	3.2	(0.9)	0.008
HDL (mmol/L), mean (SD)		1.0	(0.3)	1.0	(0.3)	0.958
Treatment persistence (days), mean (SD)		289.9	(126.8)	173	(117.7)	< 0.001

**Fig. 1:**
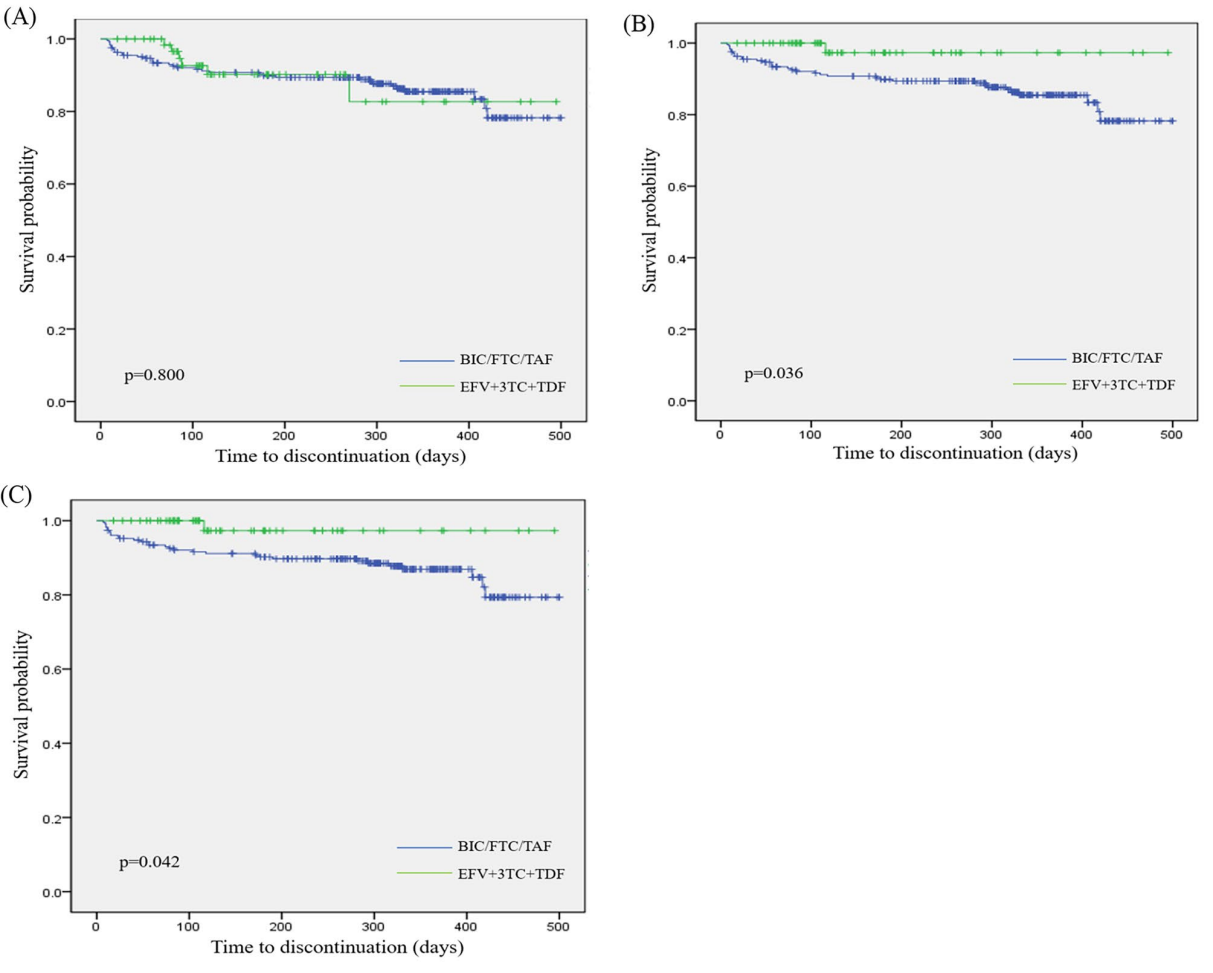
Time to discontinuation for EFV + 3TC + TDF and BIC/FTC/TAF. (**A**) All patients. (**B**) BIC/FTC/TAF patients with treatment discontinuation due to economic reason excluded. (**C**) Both BIC/FTC/TAF patients with treatment discontinuation due to economic reason and EFV + 3TC + TDF patients with a viral load > 500, 000 copies/mL excluded.

The time to discontinuation for BIC/FTC/TAF vs. EFV + 3TC + TDF in all patients was shown in Fig. 1. No significant difference was shown in the time to discontinuation between BIC/FTC/TAF and EFV + 3TC + TDF (p = 0.800). Nevertheless, after excluding BIC/FTC/TAF patients with treatment discontinuation due to economic reason, the Kaplan-Meier estimates showed that BIC/FTC/TAF group had a significantly longer time to treatment discontinuation than EFV + 3TC + TDF group (p = 0.036). After the adjustment of age group, gender, baseline CD4 count group and baseline viral load group, EFV + 3TC + TDF patients had a significantly higher risk of discontinuation than BIC/FTC/TAF patients (hazard ratio [HR] = 11.1, 95% CI = 1.3–93.2). After further removing the patients with a viral load > 500, 000 copies/mL in EFV + 3TC + TDF group, the subgroup analysis showed similar results in both Kaplan-Meier estimates (p = 0.042) and cox model (HR = 10.1, 95% CI = 1.2–84.1).

The reasons for the treatment discontinuation cases in BIC/FTC/TAF group and EFV + 3TC + TDF group were shown in Table 2. Among the 6 treatment discontinuation cases in BIC/FTC/TAF group, only 1 treatment discontinuation case was due to dyslipidemia, while majority (79.4%) of the treatment discontinuation cases were due to clinical reasons in EFV + 3TC + TDF group. Dizziness and/or sleep disturbance (40.9%), rash (22.7%), and hepatic impairment (rash and hepatic impairment: 13.6%, hepatic impairment alone: 9.1%) were the main adverse event for treatment discontinuation in EFV + 3TC + TDF group. The economic reason of the treatment discontinuation in BIC/FTC/TAF group and EFV + 3TC + TDF group was different. In EFV + 3TC + TDF group, the economic reason for treatment discontinuation was the affordable economic cost of patent drugs with better safety and efficacy profile after included in NRDL. Nevertheless, even partially reimbursed in NRDL, BIC/FTC/TAF might still imposed unaffordable economic burden to some patients, who switched from BIC/FTC/TAF to cheaper or free ART.

**Table 2:** Reasons for the treatment discontinuation cases in BIC/FTC/TAF group and EFV + 3TC + TDF group

Reason	EFV + 3TC + TDF (N = 34)		BIC/FTC/TAF (N = 6)	
	N	%	N	%
**Clinical reason**	**27**	**79.4%**	**1**	**16.7%**
Adverse event	22	64.7%	1	16.7%
Depression	1	4.5%^	0	0.0%
Dizziness and/or sleep disturbance	9	40.9%	0	0.0%
Rash	5	22.7%	0	0.0%
Rash and hepatic impairment	3	13.6%	0	0.0%
Hepatic impairment	2	9.1%	0	0.0%
Renal impairment	2	9.1%	0	0.0%
Dyslipidemia	0	0.0%	1	100%
Treatment failure due to drug resistance	5	14.7%	0	0.0%
**Economic reason**	**7**	**20.6%**	**5**	**83.3%**
Switch to BIC/FTC/TAF	6	17.6%	0	0.0%
Switch to EVG/c/FTC/TAF	1	2.9%	4	66.7%
Switch to EFV + 3TC + TDF	0	0.0%	1	16.7%

^ In the calculation of the percentage of each type of adverse event, the nominator is the total number of the adverse events in EFV + 3TC + TDF (N = 22) or BIC/FTC/TAF (N = 1) group, respectively.

*Treatment failure defined as viral load > 200 copies/mL 24 weeks after treatment initiation.

## Discussion

To our knowledge, this is the first study to evaluate the persistence of BIC/FTC/TAF and EFV + 3TC + TDF in real-world setting in China. Our study showed that after excluding the economic burden between ARTs, BIC/FTC/TAF had significant better persistence compared with EFV + 3TC + TDF, which is consistent with previous real-world studies in western countries [10, 11]. The treatment discontinuation of EFV + 3TC + TDF group is mainly due to safety reason, which is also shown in other studies reporting the neuropsychiatric side effects related with EFV [14–16]. Our study result is consistent with previous study findings showing EFV-based regimen to increase the treatment discontinuation risk due to neuropsychiatric side effects [17–19]. HIV patients in China has a high prevalence rate of depression (> 60%) and anxiety (> 40%), which could be explained by the psychological burden at diagnosis, the aversive symptoms, the stigma and guilt of the infection, and the prejudice and misconceptions about HIV [20, 21]. Therefore, ART with better safety profile should be recommended. Nevertheless, the study result is different from a prospective cohort study result published recently to evaluate the EFV discontinuation due to neuropsychiatric adverse events in China [22]. The China study showed EFV-based regimens (mainly EFV + 3TC + TDF) associated with a low risk of discontinuation due to neuropsychiatric adverse events in treatment-naïve HIV patients. One possible reason could be the higher baseline viral load in the EFV group in our study (mean HIV-1 RNA level: 50,119 [10^4.7] copies/mL) than that in the published study (mean HIV-1 RNA level: 19,175 copies/mL).

TDF related side effect is another contributor of treatment discontinuation due to safety concerns in the EFV + 3TC + TDF group. The finding is inconsistent with previous studies. Moreover, though there is a large number of HIV patients with TDF-based regimens in China, the benefits in renal functions of switching from TDF to TAF are supported by previous studies in both Western and Asian HIV patient population [23–27]. In the Swiss HIV cohort study, switching from TDF to TAF showed an improvement in eGFR and proteinuria in patients with renal dysfunction [23]. In the Japan studies, TDF patients with poor renal function could benefits from switching to TAF [24, 25].

Our study finding about the treatment discontinuation due to drug resistance in EFV + 3TC + TDF group is consistent with the previous studies about the drug resistance to free NRTI and NNRTI in China [28–34]. Drug resistance is a big threat to HIV patients in China, especially in HIV patients not taking drug resistance test before treatment initiation. Since drug resistance test is not mandatory before ART initiation and the expenditure is out-of-pocket, HIV patients may choose to initiate ART without taking drug resistance test. Although the overall prevalence of transmitted drug resistance (TDR) is low in China, high TDR rates to NNRTI in certain areas and an increasing TDR rate since 2010 have been reported [29–31]. Meanwhile, acquired drug resistance (ADR) is the main driver for treatment failure and ADR rate to NNRTI or NRTI is reported to be > 50% among treatment-failure HIV patients [32, 33]. Another study conducted in Hunan Province showed that the primary drug resistance rate of EFV and 3TC was 5.6% and 3.3%, respectively, which is higher than that reported in the EFV + 3TC + TDF group in this study [35]. The underestimated drug resistance rate could be explained by the delayed drug resistance test. In clinical practice, HIV patients would be recommended to take the drug resistance test if their viral load is not under control after taking the treatment regimens for at least half a year. Since no routine drug resistance tests performed after taking the treatment regimens, HIV patients treated with EFV + 3TC + TDF could already have the drug resistance to the treatment without knowing it. Therefore, considering the threat of drug resistance to the free NRTI and NNRTI, HIV drugs with higher resistance barrier needs to be prioritized.

The study shows BIC/FTC/TAF has significantly longer persistence compared with EFV + 3TC + TDF; however, the affordability of BIC/FTC/TAF is still a concern for HIV patients in China. After government reimbursement (≥ 70%), the out-of-pocket expenditure of BIC/FTC/TAF could still be a barrier for HIV patients, especially in HIV patients with low socio-economics status or in rural areas. Previous studies have shown that even in HIV/AIDS patient with the free ART, the out-of-pocket expenditure is still high because of the other costs related with laboratory tests, examinations, medical service and drugs for opportunistic infections, which are not covered by the government [36, 37]. A study conducted in Nantong in 2017 shows the annual hospitalization expense per HIV patient with free ART is CNY 5,454, which is slightly higher than the annual out-of-pocket expenditure of BIC/FTC/TAF, indicating a potential heavy economic burden to HIV patients taking BIC/FTC/TAF [36]. Considering the large number of HIV patients with low socio-economic status or in rural area, additional financial support needs to be prioritized to help HIV patients have access to drugs with better safety and efficacy profile.

The strength of the study includes the most recent clinical outcomes of treatment options in China. There are several limitations in our study. Firstly, since the study period is from January 1st, 2021 to July 31st, 2022, the follow-up period was relatively short and the sample size was small. The long-term persistence of BIC/FTC/3TC may need to be further assessed with a larger sample size. Secondly, data of the HIV patients in the study were collected from a single hospital in Hunan Province and therefore the study findings might not be generalized to HIV patients in cities/provinces in China. Thirdly, some variables were adjusted in the cox model, confounding effects or selection bias could not be completely ruled out.

## Conclusions

In summary, our study demonstrated the longer persistence of BIC/FTC/TAF compared with EFV + 3TC + TDF in HIV patients in Hunan Province in China. Given the 95-95-95 goals, the high burden of HIV/AIDS in China highlight the important to select treatment regimens with better safety and efficacy profiles.

## Data Availability

The data that support the findings of this study are available from the corresponding author on reasonable request. Participant data without names and identifiers will be made available after approval from the corresponding author. After publication of study findings, the data will be available for others to request. The research team will provide an email address for communication once the data are approved to be shared with others.

## Abbreviations

ADR: acquired drug resistance
ART: antiretroviral therapy
BIC/TAF/TAF: bictegravir/emtricitabine/tenofovir
CI: Confidence interval
CDC: Center for Disease Control and Prevention
DTG/ABC/3TC: dolutegravir/abacavir/lamivudine
DOR/TDF/3TC: doravirine/tenofovir/lamivudine
EFV + 3TC + TDF: efavirenz + lamivudine + tenofovir
EVG/c/FTC/TAF: elvitegravir/cobicistat/emtricitabine/tenofovir alafenamide
HR: hazard ratio
INSTI: integrase strand transfer inhibitor
MTR: multiple tablet regimens
NFATP: the national free ART program
NRTI: nucleoside reverse transcriptase inhibitors
NNRTI: non-nucleoside reverse transcriptase inhibitor
PI: protease inhibitor
SD: standard deviation
STR: single-table regimen
TDR: transmitted drug resistance
